# The systemic immune-inflammation index was non-linear associated with all-cause mortality in individuals with nonalcoholic fatty liver disease

**DOI:** 10.1080/07853890.2023.2197652

**Published:** 2023-04-13

**Authors:** Enfa Zhao, Yiping Cheng, Chunxiao Yu, Huijie Li, Xiude Fan

**Affiliations:** a Department of Ultrasound, The First Affiliated Hospital of Anhui Medical University, Hefei, China; b Department of Endocrinology, Shandong Provincial Hospital Affiliated to Shandong First Medical University, Jinan, China; c Shandong Clinical Research Center of Diabetes and Metabolic Diseases, Jinan, China; d Shandong Key Laboratory of Endocrinology and Lipid Metabolism, Jinan, China; e Department of Statistics and Medical Records Management, Shandong Provincial Hospital Affiliated to Shandong First Medical University, Jinan, China

**Keywords:** Immune-inflammation index, NAFLD, non-linear, mortality

## Abstract

**Objective:**

Systemic immune-inflammation index (SII), a novel inflammatory indicator based on platelets, neutrophils and lymphocytes, has been shown to be associated with prognostic value in several solid tumors. However, its prognostic value in nonalcoholic fatty liver disease (NAFLD) has not been reported yet. Therefore, the present study aimed to investigate the prognostic value of SII in individuals with NAFLD.

**Methods:**

Data was collected from the 2005 to 2014 National Health and Nutrition Examination Survey (NHANES, https://www.cdc.gov/nchs/nhanes/index.htm), and vital status was derived from the National Death Index (NDI) up to 31 December 2015. NAFLD was diagnosed based on Hepatic Steatosis Index (HSI). Multivariate Cox regression and Kaplan–Meier survival curves were performed to measure the hazard ratios (HRs) and 95% confidence interval (CI). Our study investigated the relationship between SII and all-cause mortality by using two-part linear regression models with penalized splines, as well as Cox models with penalized splines.

**Results:**

A total of 10,787 NAFLD participants (44.14% men) aged ≥20 years old were enrolled. There were 776 deaths from all causes after a mean follow-up period of 5.6 years. According to the full adjusted Cox regression analysis, the low log_2_-SII group (quartile 1) and the highest log_2_-SII group (quartile 4) were significantly associated with increased mortality from all causes (aHR =1.86; 95% CI: 1.47–2.37; *p* < 0.0001). After controlling for confounders, an increase in log_2_-SII was associated with an increased all-cause mortality risk of 41% for every unit raised (aHR = 1.41; 95% CI: 1.26–1.57; *p* < 0.0001). After adjusting for multiple potential confounders, the association between log_2_-SII and all-cause mortality was nonlinear, and the threshold value was 8.8. There was no association between an increase of one unit in log_2_-SII and all-cause mortality below the threshold (aHR = 0.90, 95% CI: 0.71–1.15, *p* = 0.419). However, a higher log_2_-SII was associated with a higher risk of death from any cause when it exceeded the threshold (aHR = 1. 73, 95% CI: 1.49–2.02, *p* < 0.001).

**Conclusion:**

Based on a study of US NAFLD patients, it was found that the baseline log_2_-SII is associated with all-cause mortality. Elevated SII is associated with poor survival among NAFLD patients.

**KEY MESSAGES**
1. Using a large nationally representative survey of individuals among US adults, the study demonstrated that log_2_-SII was J-shaped and associated with all-cause death among individuals with NAFLD.
2. Spline analyses demonstrated that the association between log_2_-SII and all-cause mortality was non-linear after adjusting for multiple potential confounders, and the threshold value was 8.8.
3. Higher log_2_-SII associated with poor survival in NAFLD.

## Introduction

The term nonalcoholic fatty liver disease (NAFLD) refers to a condition in which there is significant lipid accumulation in the liver without evidence of heavy alcohol consumption, viral infection or other specific causes [1]. NAFLD counts for the most prevalent chronic liver disorders worldwide and it presents with different phenotypic aspects ranging from simple steatosis to inflammation, nonalcoholic steatohepatitis (NASH), liver cirrhosis, or even hepatocellular carcinoma (HCC) [2,3]. The detailed pathophysiology of NAFLD is complicated and involves heterogeneous exogenous and endogenous factors, which involved lifestyle, nutritional factors, lipogenesis, cell death, insulin resistance, chronic low-grade inflammatory response and an altered gut microbiome [4,5]. These facts focused on the induction of systemic chronic organ inflammation, which is highly associated with many features of NAFLD. Furthermore, inflammation of the liver is primarily caused by the immune system. Due to oxidative stress, NAFLD may progress to hepatic fibrosis when Kupffer cells produce higher levels of the proinflammatory cytokine TNF-α [6]. With hepatocyte injury, activated Kupffer cells speed the secretion of proinflammatory cytokines including IL-6, TNF-α and IL-1b, which exacerbate hepatocyte injury and cell death, mainly via apoptosis [7]. Hepatic lipids trigger inflammation through TNF-α and IL-6 activation [8]. Historically, the liver has been considered a central immunological organ and is involved in the maintenance of immune homeostasis [9]. Thus, a disturbance of immune homeostasis due to different liver conditions, including NAFLD, will affect both innate and adaptive immunity, thus causing various liver disorders to develop [10,11]. Hepatocytes with NAFLD are exposed to oxidative stress and inflammation due to lipid buildup, which can progress to cirrhosis. All this evidence shows a link between inflammation and NAFLD. Therefore, a clear understanding of the possible relationship between NAFLD and inflammation is necessary to develop an effective treatment strategy for the prevention and progression of NAFLD.

Recently, the systemic immune-inflammation index (SII), a simple and robust index, has been developed as a prognostic predictor of various cancers and inflammatory diseases, which included hepatic steatosis [12,13]. According to three inflammatory biomarkers, including platelets, neutrophils and lymphocytes, SII was calculated as platelets × neutrophils/lymphocytes, which could provide a comprehensive reflection of the local immune status and systemic inflammation in the whole body at the same time [14]. The SII index was originally applied to evaluate the prognosis of individuals with solid cancers and now is adapted to properly depict inflammation status [15]. The immune-inflammatory response has been identified as being involved in multiple disease processes. As a strong predictive factor, SII has been found to be superior to conventional risk factors in the prediction of major cardiovascular events in patients with coronary heart disease after coronary intervention [16]. Furthermore, higher SII is correlated with an increased risk of peripheral arterial disease [17], isolated coronary artery ectasia [18], arteriosclerotic cardiovascular disease [19], urinary albuminuria excretion [15], diabetic kidney disease [20], diabetic depression [21] and kidney stones in US adults aged less than 50 [22]. Moreover, a recent study demonstrated a non-linear association between SII and hyperlipidemia [23], and a U-shaped correlation between the SII index and all-cause, cardiovascular disease and cancer-related mortality was observed in patients with cardiovascular disease in the general population of the United States [24]. All these pieces of evidence indicate that SII may also be potentially related to NAFLD. As for liver diseases, the liver becomes inflamed when macrophages, T lymphocytes, neutrophils and DCs infiltrate [25]. Since immune activation, inflammation and environmental factors are all associated with NAFLD pathogenesis [26], we hypothesized that an increased SII will be associated with a higher possibility of death in individuals with NAFLD. Until now, a study investigating the prognostic role of SII in NAFLD has not yet been conducted. To fill the knowledge gaps, in this study, we assessed the associations of the SII index with all-cause mortality among US adults with NAFLD in a large, nationally representative sample. Additionally, we used two-piecewise linear regression with a smoothing function to test whether log_2_-SII had any threshold effect on all-cause mortality.

## Materials and methods

### Sample sources and definition of NAFLD and SII

The NHANES database, conducted by the National Center for Health Statistics (NCHS), is a nationwide survey to evaluate the health issue of US residents. It intended to supervise the health and nutritional status of civilian, noninstitutionalized US inhabitants in the US population using a complex, multistage design with data released in 2-year cycles. Data and study design details for the NHANES can be found online at https://www.cdc.gov/nchs/nhanes/. Using household interviews, clinical examinations and mobile examination centers (MECs), baseline demographic and health-related questions are collected. Neutrophil, lymphocyte and platelet counts were performed through an automated hematology analyzing device (Coulter^®^ DxH 800 analyzer) to complete the blood count and expressed as ×10^3^ cells/μl. SII was defined as platelet count × neutrophil count/lymphocyte count according to previous studies [14,15,27]. NIHNES received approval from the National Center for Health Statistics’ institutional review board and all participants provided written informed consent.

**Figure 1.**
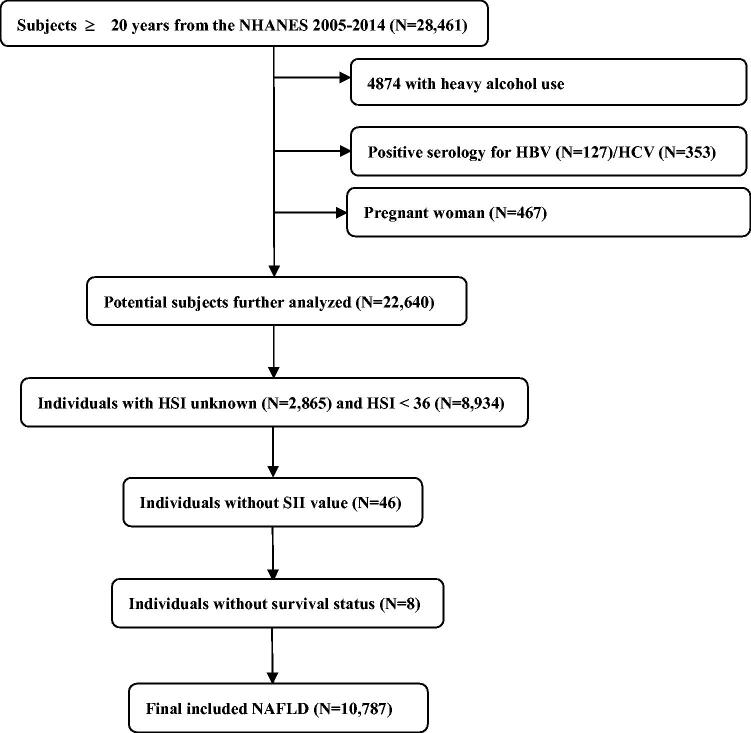
The visual flow-process diagram of this study.

The NHANES data set consisting of 28,461 participants ≥20 years old between 2005 and 2014 was used for analyses. Among the eligible adults, we first excluded 4874 individuals with heavy alcohol use (≥3 drinks/day for females, ≥4 drinks/day for males), hepatitis B virus (*n* = 127), hepatitis C virus (*n* = 353), or during pregnancy (*n* = 467). Next, NAFLD was identified using the Hepatic Steatosis Index (HSI) formula in the remaining individuals as follows: HSI = 8 × ALT/AST + BMI (+2, if DM; +2, if female) [28]. The presence of NAFLD was identified when the HSI score was >36 according to the previously validated cut-off [28,29]. Among 22,640 individuals, we further removed participants without HSI value (*n* = 2,865) and HSI <36 (*n* = 8,934). Finally, individuals without SII value (*n* = 64) or survival status (*n* = 8) were further eliminated. As a result, a total of 10,787 NAFLD were analyzed (Figure 1). The Fibrosis-4 index (FIB-4), which is a non-invasive fibrosis score, was adapted to qualify the possibility of liver fibrosis, and a score of above 2.67 was classified as advanced fibrosis [30].

### Assessment of covariates

Baseline characteristics, and socio-demographics, including age, sex (male, female), waist circumference, race (Mexican American, non-Hispanic Black, non-Hispanic White and other), family poverty income ratio (PIR), marital status (married, unmarried), smoking habits (never, former, now), body mass index (BMI), education level (less than high school, high school and high school above), and history of asthma, diabetes, cancer, hypertension and cardiovascular disease (CVD) were provided by the individuals during the household interviews. Those who had been told they had coronary heart disease, congestive heart failure, a heart attack, a stroke, or angina were considered to have a history of CVD. BMI was grouped into normal (18.5 ∼ 23 kg/m^2^), overweight (23 ∼ 25 kg/m^2^), and obese (≥25 kg/m^2^). A diagnosis of diabetes is based on: (1) a self-reported diagnosis of diabetes by a medical professional; (2) self-reported use of anti-diabetic medications; or (3) fasting blood glucose levels >126 mg/dL or HbA1c ≥6.5%. In addition to these laboratory indicators, other laboratory indicators were selected based on the clinical significance or literature evidence. On the CDC’s website, https://www.cdc.gov/nchs/nhanes/, you can find a detailed description of laboratory testing procedures and quality control strategies. Indicators used in the laboratory included total cholesterol (mg/dL), alanine transaminase (ALT, IU/L), glycated hemoglobin A1c (HbA1c, %), serum creatinine (mg/dL), aspartate transaminase (AST, IU/L) and HDL cholesterol (mg/dL).

### Exposure and clinical outcome

In the present study, SII was taken into account as an exposure variable. Mortality data were extracted from the 2005 to 2014 NHANES public-use linked mortality files. Online access to detailed mortality records is available to everyone (https://www.cdc.gov/nchs/datalinkage/mortality-public.htm). Deaths due to all causes constituted the primary endpoint. The follow-up duration was calculated as the time from enrollment (date of interview) to mortality for censoring.

### Statistical analysis

A multistage sample design was utilized in NHANES, so weights were used appropriately for statistical analysis. Survey-weighted means (95% confidence intervals) were used for continuous variables, and survey-weighted percentages were used for categorical variables. Additionally, since SII were right-skewed distributed, thus data were log_2_-transformed before conducting statistical analysis. A log_2_-SII value was calculated for each patient and divided into four quartiles, with the lowest quartile used as a reference. As a comparison to those in quartile 1, we computed the hazard ratios (HRs) and 95% confidence intervals (CIs) for individuals in quartile 2, quartile 3 and quartile 4. An additional analysis was conducted using log_2_-SII as a continuous variable. The survival analysis for the effect of log_2_-SII quartiles was explored using crude Kaplan–Meier curves. In order to estimate the main effects of log_2_-SII on patients’ survival, multiple multivariate Cox regression models were used. In model 1, none variables were adjusted. In model 2, we adjusted for age (years), sex (male or female), BMI, waist circumference, race (Mexican American, non-Hispanic Black, non-Hispanic White and other), marital status (married, unmarried), education level (less than high school, high school and high school above), family poverty income ratio and smoking (never, former, now). In model 3, we further adjusted for asthma, cancer, diabetes, hypertension, creatinine, cholesterol, ALT, AST, HbA1c, HDL and creatinine. The variance inflation factor (VIF) was used to check for multi-collinearity, and variables with a VIF greater than 10 were excluded from the model. An analysis of two-piecewise linear regression with a smoothing function was further used to test whether log_2_-SII had any threshold effect on all-cause mortality. It was determined that a threshold level (or turning point) could be achieved using trial and error; turning points were selected along a predetermined interval, and the turning point that gave the greatest likelihood of the model was identified. We also compared a one-line linear regression model with a two-piecewise linear model using the log-likelihood ratio. Stratified analyses were implemented by gender (male, female), age (<60, ≥60 years), smoking status (never, former, now), education level (less than high school, high school, high school above), hypertension (yes, no), diabetes (yes, no), CVD (yes, no), asthma (yes, no), cancer (yes, no), marital status (married, unmarried), BMI (normal, overweight, obese) and advanced fibrosis (yes, no). We examined the significance of interactions between continuous log_2_-SII and multiple stratification variables using the *p*-values for the product terms.

We tested the robustness of our results by conducting a number of sensitivity analyses. Firstly, as a sensitivity analysis, NAFLD deaths within 2 years of follow-up were excluded in order to eliminate possible reverse causality. Second, as no variables had missing values greater than 5%, the missing values were accounted for using a multiple imputation technique based on 10 replications. Then 10 complete datasets were analyzed for sensitivity, and the results were pooled. Furthermore, the problem of unmeasured confounding frequently arises in observational epidemiological studies. To examine the impact of such confounding on our main findings, we performed a formal sensitivity analysis. Using the E-value algorithm, we quantified the minimum degree of correlation between unmeasured predictors and outcomes or exposures that would fully explain associations between SII (the exposure) and all-cause death (the outcome) [31]. All analyses were conducted with R (http://www.R-project.org; version 3.4.3) and EmpowerStats software (www.empowerstats.com, X&Y solutions, Inc., Boston, MA, USA).

## Results

### Characteristics of NAFLD participants

A total of 10,787 NAFLD adults were included with a mean age of 51.9 ± 16.4, of which 4,761 (44.1%) were males, and 1868 (17.3%) were Mexican American. A table displaying the weighted demographic baseline characteristics of included patients is presented in Table 1. The weighted mean (95% confidence interval) of log_2_-SII was 8.96 (8.93, 8.99). Participants with high log_2_-SII quartiles tend to be having higher BMI, higher waist circumference, more female, more non-Hispanic White and more unmarried, more now smoking, more high school level, more history of asthma, diabetes, cancer and hypertension; while tend to have lower AST and ALT.

**Table 1.** Baseline characteristics of NAFLD individuals according to systemic immune-inflammation index quartiles, weighted.

	Q1 (<8.4)	Q2 (8.4–8.9)	Q3 (8.9–9.4)	Q4 (>9.4)	*p* For trend
Age (years)	50.19 (49.28, 51.10)	50.08 (49.33, 50.84)	49.64 (48.94, 50.35)	50.52 (49.58, 51.45)	0.7035
BMI (kg/m^2^)	32.39 (32.13, 32.65)	32.55 (32.26, 32.84)	33.13 (32.85, 33.41)	34.74 (34.36, 35.12)	<0.0001
Waist circumference (cm)	107.03 (106.33, 107.73)	107.67 (106.99, 108.35)	108.88 (108.26, 109.51)	111.54 (110.73, 112.35)	<0.0001
Family poverty income ratio	2.90 (2.79, 3.01)	3.07 (2.98, 3.17)	3.02 (2.91, 3.12)	2.94 (2.82, 3.07)	0.9219
Total cholesterol (mg/dL)	196.47 (194.56, 198.38)	198.02 (195.83, 200.21)	198.70 (196.71, 200.68)	196.71 (194.74, 198.69)	0.8212
ALT(U/L)	29.60 (28.76, 30.44)	29.18 (28.43, 29.93)	28.52 (27.57, 29.48)	26.53 (25.65, 27.42)	<0.0001
AST(U/L)	26.53 (26.00, 27.06)	25.56 (25.19, 25.93)	25.44 (24.88, 26.01)	24.60 (24.02, 25.17)	<0.0001
HbA1c (%)	5.84 (5.79, 5.88)	5.79 (5.73, 5.84)	5.78 (5.74, 5.83)	5.89 (5.83, 5.94)	0.1711
HDL cholesterol (mg/dL)	48.51 (47.74, 49.29)	47.94 (47.27, 48.61)	48.22 (47.62, 48.81)	48.89 (48.17, 49.61)	0.324
Creatinine (mg/dL)	0.92 (0.91, 0.93)	0.90 (0.89, 0.92)	0.90 (0.88, 0.91)	0.92 (0.90, 0.94)	0.9956
Sex					
Female	48.99 (46.66, 51.32)	51.63 (49.16, 54.09)	53.10 (50.81, 55.38)	61.51 (59.07, 63.88)	<0.0001
Male	51.01 (48.68, 53.34)	48.37 (45.91, 50.84)	46.90 (44.62, 49.19)	38.49 (36.12, 40.93)	<0.0001
Race					
Mexican American	9.30 (7.50, 11.49)	8.77 (6.96, 10.99)	9.22 (7.45, 11.36)	7.99 (6.33, 10.04)	0.1069
Non-Hispanic Black	22.08 (18.83, 25.71)	11.65 (9.89, 13.69)	9.73 (8.23, 11.48)	9.11 (7.56, 10.93)	<0.0001
Non-Hispanic White	58.47 (53.68, 63.11)	67.91 (64.27, 71.33)	70.87 (67.08, 74.38)	73.13 (69.43, 76.53)	<0.0001
Other	10.15 (8.38, 12.24)	11.67 (10.04, 13.53)	10.18 (8.42, 12.26)	9.78 (8.20, 11.62)	0.3524
Education					
Less than high school	20.24 (17.99, 22.69)	17.11 (15.16, 19.24)	16.30 (14.29, 18.54)	17.73 (15.61, 20.07)	0.0696
High school	23.20 (21.09, 25.46)	21.86 (19.51, 24.41)	23.42 (21.11, 25.89)	25.81 (23.78, 27.95)	0.0274
High school above	56.56 (53.38, 59.69)	61.03 (58.18, 63.82)	60.28 (57.07, 63.41)	56.46 (53.63, 59.25)	0.7192
Marital status					
Unmarried	31.98 (29.60, 34.46)	29.09 (26.93, 31.36)	31.85 (29.89, 33.87)	36.44 (33.97, 38.99)	0.0005
Married	68.02 (65.54, 70.40)	70.91 (68.64, 73.07)	68.15 (66.13, 70.11)	63.56 (61.01, 66.03)	0.0005
Smoking					
Never	60.4 (57.7, 63.0)	59.5 (56.9, 62.0)	58.7 (56.0, 61.4)	56.0 (53.6, 58.4)	0.0112
Former	26.0 (23.7, 28.3)	28.0 (25.6, 30.4)	26.4 (24.3, 28.5)	27.6 (25.1, 30.2)	0.5808
Now	13.6 (11.9, 15.5)	12.6 (11.1, 14.2)	14.9 (13.3, 16.8)	16.4 (14.7, 18.2)	0.0034
Asthma					
No	85.47 (83.65, 87.11)	86.86 (85.39, 88.20)	83.77 (81.72, 85.62)	82.09 (79.98, 84.02)	0.0023
Yes	14.53 (12.89, 16.35)	13.14 (11.80, 14.61)	16.23 (14.38, 18.28)	17.91 (15.98, 20.02)	0.0023
CVD					
No	88.99 (87.50, 90.32)	90.49 (89.29, 91.57)	90.74 (89.44, 91.89)	87.49 (86.06, 88.79)	0.1264
Yes	11.01 (9.68, 12.50)	9.51 (8.43, 10.71)	9.26 (8.11, 10.56)	12.51 (11.21, 13.94)	0.1264
Diabetes					
No	80.89 (79.00, 82.66)	82.42 (80.66, 84.05)	81.18 (79.45, 82.79)	77.52 (75.33, 79.57)	0.0066
Yes	19.11 (17.34, 21.00)	17.58 (15.95, 19.34)	18.82 (17.21, 20.55)	22.48 (20.43, 24.67)	0.0066
Cancer					
No	90.81 (89.10, 92.28)	90.87 (89.46, 92.11)	89.85 (88.10, 91.37)	88.42 (86.83, 89.83)	0.0168
Yes	9.19 (7.72, 10.90)	9.13 (7.89, 10.54)	10.15 (8.63, 11.90)	11.58 (10.17, 13.17)	0.0168
Hypertension					
No	53.81 (51.56, 56.03)	54.07 (51.42, 56.71)	52.31 (50.28, 54.33)	48.25 (45.46, 51.04)	0.0007
Yes	46.19 (43.97, 48.44)	45.93 (43.29, 48.58)	47.69 (45.67, 49.72)	51.75 (48.96, 54.54)	0.0007
Advanced fibrosis					
No	98.33 (97.76, 98.75)	97.22 (96.10, 98.02)	97.15 (96.00, 97.99)	96.00(94.80, 96.93)	0.0001
Yes	1.67 (1.25, 2.24)	2.78 (1.98, 3.90)	2.85 (2.01, 4.00)	4.00(3.07, 5.20)	0.0001

### Association between log_2_-SII and mortality

**Figure 2.**
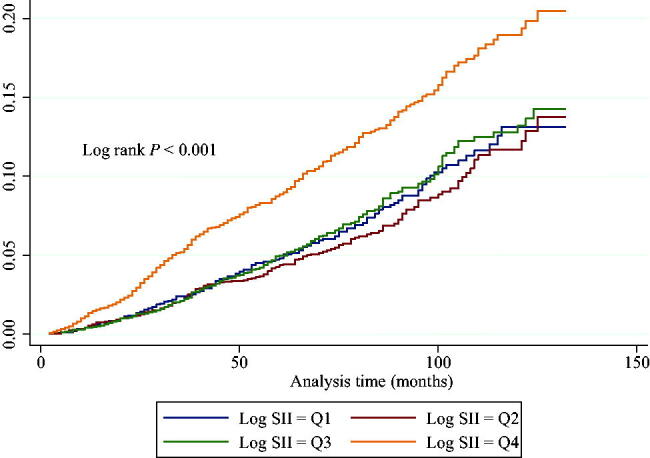
Cox cumulative hazard function for all-cause death stratified by log_2_-SII quartiles. Participants with the highest concentrations of log_2_-SII (quartile 4) exhibited remarkably worse outcome compared to individuals with lower log_2_-SII concentrations.

Following a follow-up period of 67.8 months (range: 2–132 months), there were 776 (7.2%) mortality events. In unadjusted Kaplan–Meier analyses, it was demonstrated that individuals with log_2_-SII in the highest quartile (Q4) demonstrated the highest cumulative incidence of mortality due to all-cause during follow-up compared to individuals with other quartiles (Log-rank *p* < 0.001, Figure 2). A crude and fully adjusted association between log_2_-SII and all-cause death is shown in Table 2. In the unadjusted Cox regression model, when individuals in the Q1 were used as a reference, those in the highest quartiles presented with a higher risk of death (HR = 1.69, 95% CI = 1.39–2.06; *p* < 0.001). These associations remained essentially unchanged in the minor adjusted model (aHR = 1.86, 95% CI = 1.50–2.30; *p* < 0.001) and the fully adjusted model (aHR = 1.86, 95% CI = 1.47–2.37; *p* < 0.001). When treating log_2_-SII as a continuous variable, the results remained the same. In a fully adjusted model, after controlling for confounders, an increase in log_2_-SII was associated with an increased all-cause mortality risk of 41% for every unit raised (aHR = 1.41; 95% CI: 1.26–1.57; *p* < 0.0001).

**Table 2.** Associations of the SII with all-cause mortality in individuals with NAFLD.

	Non-adjusted	Adjust I	Adjust II
Log SII	1.45 (1.31, 1.59); <0.0001	1.43 (1.29, 1.58); <0.0001	1.41 (1.26, 1.57); <0.0001
Log SII quartile			
Q1	1 (Reference)	1 (Reference)	1 (Reference)
Q2	0.91 (0.73, 1.14); 0.4264	1.02 (0.80, 1.30); 0.8752	1.03 (0.79, 1.35); 0.8390
Q3	1.04 (0.84, 1.29); 0.7153	1.17 (0.92, 1.48); 0.1960	1.15 (0.89, 1.50); 0.2907
Q4	1.69 (1.39, 2.06); <0.0001	1.86 (1.50, 2.30); <0.0001	1.86 (1.47, 2.37); <0.0001
*p* For trend	<0.001	<0.001	<0.001

Non-adjusted model adjust for: None.

Adjust I model adjust for: age, sex, race, BMI, waist circumference, marital status, education, smoking and family poverty income ratio.

Adjust II model adjust for: age, sex, race, BMI, waist circumference, marital status, education, smoking and family poverty income ratio, asthma, cancer, diabetes; hypertension, smoking, creatinine, cholesterol, ALT, AST, HbA1c, HDL, creatinine and advanced fibrosis.

### Non-linear relationship between log_2_-SII and mortality

**Figure 3.**
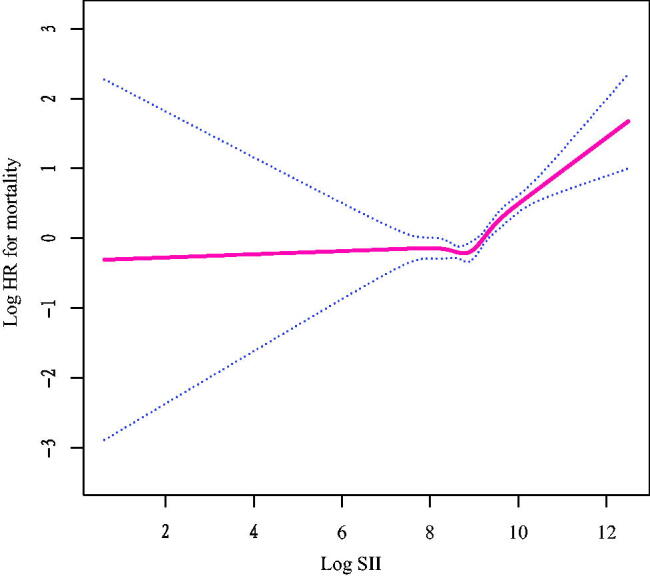
Relationship between log_2_-SII and all-cause mortality by smooth curve fitting. Adjustment for age, sex, race, BMI, waist circumference, marital status, education, smoking and family poverty income ratio, asthma, cancer, diabetes; hypertension, smoking, creatinine, cholesterol, ALT, AST, HbA1c, HDL and creatinine. The red line demonstrates the risk of mortality, and the blue dotted lines illustrate its 95% confidence interval.

The adjusted multivariate Cox regression models with penalized splines presented a nonlinear association between log_2_-SII (continuous variable) and the risk of all-cause death (*p* for nonlinearity <0.001, Figure 3). Based on threshold effect analyses, log_2_-SII was related to all-cause death in a non-linear manner when multiple confounders were taken into account, and the threshold value for this association was 8.8. (SII = 445.7). Log_2_-SII was not associated with all-cause mortality below the threshold (aHR = 0.90, 95% CI: 0.71–1.15, *p* = 0.419). However, log_2_-SII above a threshold was associated with an increased risk of all-cause death (aHR = 1.73, 95% CI: 1.49–2.02, *p* < 0.001; Table 3).

**Table 3.** Threshold analysis for the relationship between log_2_-SII and all-cause mortality in patients with NAFLD.

Models	Adjusted HR (95% CI); *p*-value
Model I	
One line slope	1.40 (1.25, 1.56); <0.001
Model II	
Turning point (K)	8.8
<8.8	0.90 (0.71, 1.15); 0.419
>8.8	1.73 (1.49, 2.02); <0.001
HR between <8.8 and >8.8	1.92 (1.37, 2.69); 0.0002
Logarithmic likelihood ratio test	<0.001

Adjust for: age, sex, race, BMI, waist circumference, marital status, education, smoking and family poverty income ratio, asthma, cancer, diabetes; hypertension, smoking, creatinine, cholesterol, ALT, AST, HbA1c, HDL, creatinine and advanced fibrosis.

### Subgroup analyses and sensitivity analyses

**Figure 4.**
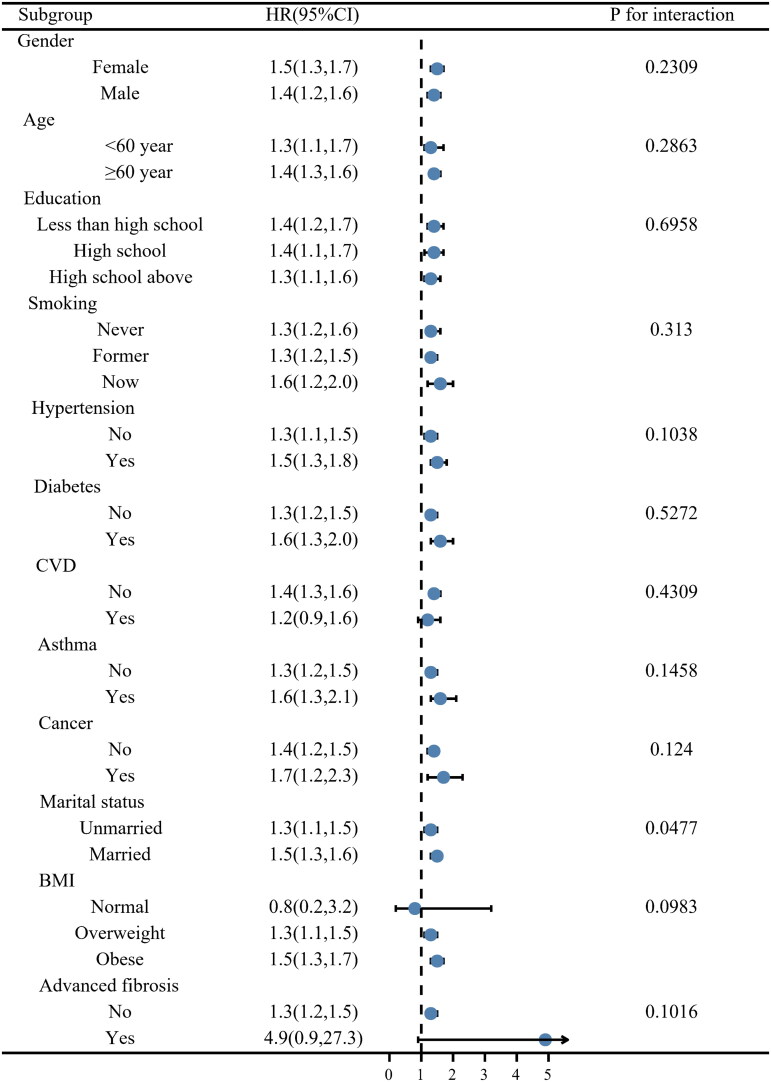
Stratification analysis of log_2_-SII with all-cause mortality in NAFLD.

In this study, a subgroup analysis was conducted to examine the robustness of the correlation between log_2_-SII and death of all causes. The results of all subgroup analyses except for normal BMI and the history of CVD subgroups also confirmed this finding (Figure 4), which indicates the robustness of our results. Statistical interaction was noticed for marital status, and the associations with all-cause death were stronger among individuals who were married (*p* = 0.0477).

After conducting a sensitivity analysis and eliminating those who died in the first 2 years, the results remained similar (Supplementary Table 1). In the fully adjusted Cox regression model, when individuals in the Q1 were used as a reference, those in the highest quartiles presented with a higher risk of death (aHR = 1.68, 95% CI = 1.32–2.14; *p* < 0.001). When treating log_2_-SII as a continuous variable, the results persisted in a fully adjusted model (aHR = 1.32; 95% CI: 1.18–1.48; *p* < 0.0001). Furthermore, we handled missing data with multiple imputations. After adjusting for multiple variables, the fundamental result was unchanged (aHR = 1.81; 95% CI: 1.48–2.21; *p* < 0.0001; Supplemental Figure 1). Moreover, to evaluate the influence of unmeasured confounding, using HRs for all-cause deaths, E-values (with their lower 95% confidence intervals) were calculated (Supplemental Figure 2). Unmeasured variables were related to both log_2_-SII and all-cause mortality by HRs of 2.45-fold; weaker confounding did not change these associations. As a result, the E-value and sensitivity analyses above confirmed the robustness of the findings.

## Discussion

As far as we know, using a representative sample of US adults with NAFLD, this study is the first to investigate the relationship between SII and the risk of all-cause death in NAFLD. Our findings demonstrated that in NAFLD defined by ultrasound, higher SII was associated with greater mortality, and this association persisted after adjustment for multiple confounding variables. In NAFLD populations, there was a J-shaped curve associated with all-cause deaths, with the threshold value being 8.8 log_2_-SII. There was no association between an increase of one unit in log_2_-SII and all-cause mortality below the threshold. However, the log_2_-SII was associated with a higher death rate from all causes above the threshold.

The most common form of chronic liver disease in the developed world is NAFLD, with a global prevalence ranging from 13 to 28% [32]. The initiation and progression of NAFLD are associated with metabolic and inflammatory disturbances and genetic and environmental characteristics that drive the persistent activation of the immune system and low-grade inflammation [33]. Liver inflammation (usually NASH) and systemic inflammation are present in 10–30% of all individuals with NAFLD and are generally followed by liver damage and subsequently liver fibrosis [34]. Based on the abovementioned background, the SII has gained increasing attention in recent years.

As SII is an indicator of the comprehensive evaluation system derives from peripheral lymphocyte, neutrophil and platelet counts, which probably reflected three pathways of thrombus formation, inflammation and adaptive immunity [35,36]. Previous works of research have reported the prognostic role of SII in various diseases. As a simple, reliable and minimally invasive biomarker, SII was first developed in 2014 and found that it can use as a powerful prognostic index in individuals with hepatocellular carcinoma [27]. A recent study demonstrated that in colorectal cancer, it provided the most accurate prediction for long-term survival compared to neutrophil–lymphocyte ratio and platelet–lymphocyte ratio [37]. For the prognostic value of SII, a possible explanation may be that in comparison with the other indicators, SII was more comprehensive at capturing the status of inflammation and immunity. As we all know, inflammation is vital to the occurrence and development process of NAFLD, which brings changes in the levels of peripheral blood leukocytes [34]. The presence of neutrophils in the liver is a hallmark of inflammation in multiple types of liver disease. Neutrophil infiltration is common in individuals with NAFLD or NASH, resulting in macrophage recruitment and cell damage from the release of reactive oxygen species and inflammatory mediators such as myeloperoxidase and elastase [38]. Under the condition of systemic inflammation, total circulating neutrophils and platelets raises while the number of lymphocytes declines. It has been proved that activated hepatic stellate cells increase the survival of neutrophils, which further contributes to liver fibrosis through the production of reactive oxygen species [39]. Nevertheless, in a recent study, neutrophils were shown to play a protective role in liver fibrosis through their complex roles in both chronic liver inflammation and fibrosis [40]. Furthermore, despite having low neutrophil and platelet counts or low blood cell counts within clinically normal ranges, many individuals continue to be at risk for cardiovascular events [41,42]. These findings together with our findings suggest SII may contribute to the survival of NAFLD. As we know, platelets play a crucial role in hemostasis as well as, in particular, in pathological conditions such as arterial thrombotic diseases. Chronic inflammation and tissue fibrosis can be triggered by activated platelets through the release of soluble mediators, such as TXA2 and TGF-β [43]. Recently, it was discovered that platelets play a crucial role in the pathogenesis of liver and systemic diseases. Compared with healthy controls, the increase of inflammatory transcripts was significant in the circulating platelets of NAFLD individuals [44]. Recent prospective studies showed that when taken daily, aspirin is associated with fewer histologic features of biopsy-proven NAFLD and NASH and a significantly lower possibility for progression to advanced fibrosis [45,46]. The role of innate and innate-like lymphocytes including the hepatic natural killer, innate lymphoid cells, natural killer T and γδT cells contributed to NASH pathogenesis and damaged hepatocytes with lipid accumulation trigger innate immune responses via multiple pathways [47]. However, it has been shown that individuals with advanced fibrosis are likely to have decreased platelets and that low platelet count (<15 × 10^−4^) has been associated with poor survival in NAFLD [48]. This may not be consistent with the results of this study. In our study, higher log_2_-SII is associated with poor survival in NAFLD. In fact, platelets play a critical role in atherogenesis, inflammation and prothrombotic potential in arterial thrombosis. Atherosclerosis occurs when active platelets interact with endothelium, leukocytes and nonactive platelets, and they contribute to the inflammatory process [49,50]. The decrease in lymphocyte count is associated with atherosclerosis progression. As the lipid core of an atherosclerotic plaque develops, it ruptures and forms thrombus [51]. All these statements demonstrate that the integrated index SII can indicate the balance between host immunity and inflammation response situation. Thus, based on the evidence above, the protective effect of aspirin against NAFLD may account for the non-linear relationship between log_2_-SII and mortality in a US NAFLD population. SII has exhibited excellent predictive power in various studies, and as a common method, SII is non-invasive and low-cost. Because of this, there is a great deal of potential for the clinical application of SII in NAFLD. Furthermore, the findings also revealed that among patients with NAFLD, a lower SII did not increase the risk of all-cause mortality, which suggests that anti-inflammation therapies should not work for lowering all-cause mortality in NAFLD patients. This may not be inconsistent with the opinion from Iran that a diet low in inflammation, evaluated by the dietary inflammatory index (DII), can help balance liver enzymes, reduce obesity and lower the risk of fatty liver disease [52]. The difference could be explained by the difference in the definition of inflammation indices, study population and endpoint events. Future studies will still need to confirm these findings.

Our research has some advantages. First, this study is strong in terms of its large sample size and representative sample selection. On the basis of a nationally representative survey of US adults, this was a prospective cohort study with large sample size. Second, reverse causality was minimized by excluding individuals who died within 2 years of the baseline interview, which did not decrease the relationship between SII and all-cause death. Third, the stable nature of our findings was further confirmed by multiple imputations. Fourth, we conducted three sensitivity analyses to verify the reliability of the main conclusions. Finally, it was possible to identify multiple confounders in NHANES, which were able to adjust for them in different models. Besides, based on sensitivity analysis via E-value, log_2_-SII had robust associations with mortality regardless of unmeasured confounding factors.

Nevertheless, this study is limited in several ways before it can be concluded. Firstly, at baseline, platelets, neutrophils and lymphocytes were measured only one time, which may fail to reveal subtle changes that may have occurred during the follow-up. Secondly, biochemical tests rather than liver biopsy are used for the diagnosis of NAFLD. Of course, hepatic steatosis can only be diagnosed by a liver biopsy. However, liver biopsy is expensive, invasive and therefore impractical for routine clinical use by patients with NAFLD in a large nationally representative survey. Besides, we cannot exclude the probability of unmeasured confounding. In addition, we failed to rule out the drug-induced hepatotoxicity in the NAFLD identification in an epidemiological survey. Thus, we were unable to determine a causal relationship between drug use history and fatty liver. Besides, may affect these measures such as aspirin use, use of non-steroid anti-inflammatory drugs or anticoagulants are not available and are not considered. However, multiple sensitivity analyses and robust results were obtained across subgroups. E value evaluating the sensitivity to potential unmeasured confounding support the robustness of the conclusion.

## Conclusion

Our study showed that higher SII levels are associated with increased mortality in NAFLD populations identified by ultrasound. This association persisted even after adjusting for multiple confounding variables. We observed a J-shaped curve associated with all-cause deaths in NAFLD populations, with a threshold value of 8.8 log_2_-SII. Below this threshold, there was no significant association between an increase of one unit in log_2_-SII and all-cause mortality. However, above the threshold, log_2_-SII was significantly associated with a higher death rate from all causes. These results suggest that SII may be a useful prognostic predictor for all-cause mortality in NAFLD populations and that maintaining SII levels below the threshold value may help to reduce the risk of mortality. A greater understanding of the effects of interfering with SII on NAFLD survival will need to be conducted in future studies.

## Supplementary Material

Supplemental Material

Supplemental Material

Supplemental Material

## Data Availability

A publicly available dataset was analyzed in this study. The National Health and Nutrition Examination Survey dataset is publicly available at https://www.cdc.gov/nchs/nhanes/index.htm.
