# Supplementary figures and images for: The systemic immune-inflammation index was non-linear associated with all-cause mortality in individuals with nonalcoholic fatty liver disease
Source: Ann Med. 2023 Apr 13;55(1):2197652. doi: 10.1080/07853890.2023.2197652 (PMC10115001; doi:10.1080/07853890.2023.2197652)

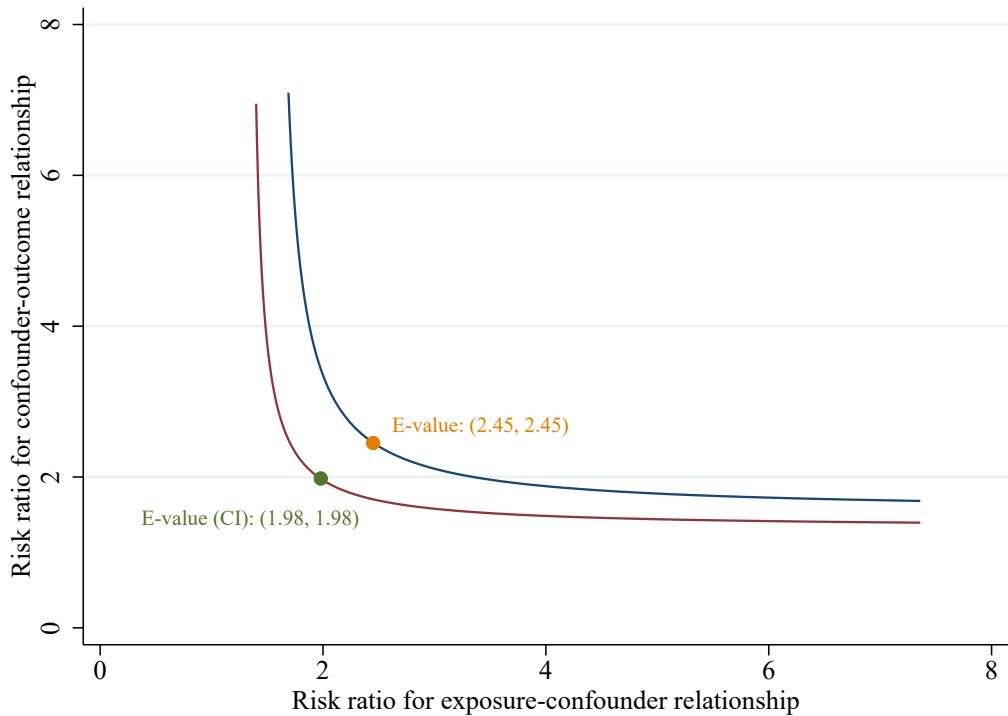

Supplement: Supplemental Material [file IANN_A_2197652_SM8068.pdf]

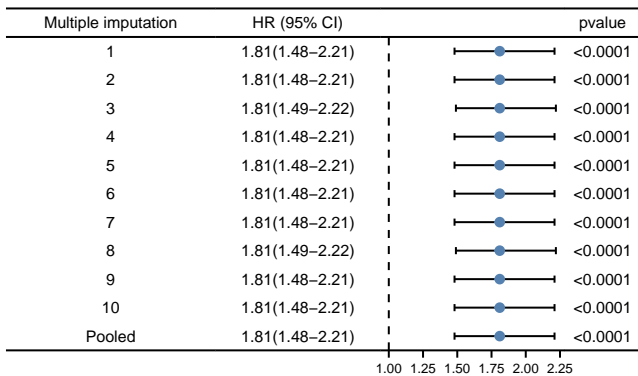

Supplement: Supplemental Material [file IANN_A_2197652_SM8067.pdf]
