# Supplementary material for: The systemic immune-inflammation index was non-linear associated with all-cause mortality in individuals with nonalcoholic fatty liver disease
Source: Ann Med. 2023 Apr 13;55(1):2197652. doi: 10.1080/07853890.2023.2197652 (PMC10115001; doi:10.1080/07853890.2023.2197652)
Supplement: Supplemental Material [file IANN_A_2197652_SM8066.docx]

Table S1 Sensitivity analyses by excluding NAFLD individuals who died in the first 2 years of follow-up

|  | Non-adjusted | Adjust I | Adjust II |
| --- | --- | --- | --- |
| Log SII | 1.33 (1.20, 1.48) <0.0001 | 1.32 (1.18, 1.47) <0.0001 | 1.32 (1.18, 1.48) <0.0001 |
| Log SII quartile |  |  |  |
| Q1 | 1 (Reference) | 1 (Reference) | 1 (Reference) |
| Q2 | 0.90 (0.70, 1.15) 0.4023 | 1.00 (0.77, 1.30) 0.9847 | 0.97 (0.74, 1.28) 0.8497 |
| Q3 | 1.05 (0.83, 1.34) 0.6669 | 1.16 (0.91, 1.49) 0.2286 | 1.16 (0.90, 1.50) 0.2617 |
| Q4 | 1.56 (1.25, 1.94) <0.0001 | 1.63 (1.29, 2.06) <0.0001 | 1.68 (1.32, 2.14) <0.0001 |

Non-adjusted model adjust for: None

Adjust I model adjust for: age, sex, race, BMI, waist circumference, marital status, education, smoking, and family poverty income ratio;

Adjust II model adjust for: age, sex, race, BMI, waist circumference, marital status, education, smoking, and family poverty income ratio, asthma, cancer, diabetes; hypertension, smoking, creatinine, cholesterol, ALT, AST, HbA1c, HDL, and creatinine
